# Who Is Watching the Children? A Quantitative Analysis of Strategies for Reconciling Work and Parenting during Lockdown in Northern Italy

**DOI:** 10.3390/ijerph182111174

**Published:** 2021-10-24

**Authors:** Barbara Plagg, Heidi Flarer, Andreas Conca, Christian J. Wiedermann, Adolf Engl, Giuliano Piccoliori, Sigrid Mairhofer, Verena Barbieri, Klaus Eisendle

**Affiliations:** 1 Institute of General Practice and Public Health, Provincial College for Health Professions Claudiana, 39100 Bolzano, Italy; heidi.flarer@eurac.edu (H.F.); christian.wiedermann@am-mg.claudiana.bz.it (C.J.W.); adolf.engl@am-mg.claudiana.bz.it (A.E.); giuliano.piccoliori@am-mg.claudiana.bz.it (G.P.); verena.barbieri@am-mg.claudiana.bz.it (V.B.); klaus.eisendle@sabes.it (K.E.); 2 Faculty of Education, Free University of Bolzano Bozen, 39100 Bolzano, Italy; 3 Department of Psychiatry, Bolzano Central Hospital, 39100 Bolzano, Italy; andreas.conca@sabes.it; 4 Department of Public Health, Medical Decision Making and HTA, University of Health Sciences, Medical Informatics and Technology, 6060 Hall in Tyrol, Austria; 5 Department of Applied Social Sciences, Munich University of Applied Sciences, 80335 München, Germany; sigrid.mairhofer@hm.edu

**Keywords:** COVID-19, lockdown, family health, public health, health inequities, education, prevention

## Abstract

(1) Background: In their efforts to contain the spread of COVID-19, most countries closed schools and kindergartens. To date, little is known about the strategies of working families reconciling work and parenting during repeated lockdown situations. (2) Methods: We performed a quantitative survey of working parents in Italy during a week of ‘hard lockdown’ in February/March 2021. (3) Results: 3725 voluntary adult participants from different households responded. Though officially not allowed, 53.4% of all participants sought help from people outside the nuclear family to bridge the situation, mostly the grandparents (79%; n = 1855). Overall, parental coping strategies included alternating working–childcare-turns with their partner (35%, n = 1316), working early in the morning or during nighttime (23%; n = 850), or leaving the children unattended (25%, n = 929). (4) Conclusions: The closure of schools/kindergartens forcefully shifts the responsibility for childcare onto the nuclear family, where new strategies arose, including health-damaging models of alternating work–childcare-shifts, ‘illegal’ involvement of third parties from outside the nuclear family, as well as neglect of age-related childcare. Our findings underline that working families need additional support strategies during repeated closure of childcare institutions to be able to reduce contact and minimize secondary damage.

## 1. Introduction

In their efforts to contain the spread of severe acute respiratory syndrome coronavirus 2 (SARS-CoV-2), most countries closed schools, kindergartens, and nurseries. Globally, schools have been fully closed for approximately half the time intended for classroom education between March 2020 and February 2021 [1]. Suspension of face-to-face instruction at school and closure of kindergartens and nurseries significantly changed the lives of preschool- or schoolchildren and their families [2,3]. The effectiveness of school closures is difficult to analyze, since it is hardly possible to disentangle the effects of each specific intervention and school and kindergarten closures were implemented along with with a range of other non-pharmacological interventions (NPIs).

Early evidence has been provided that the pandemic represents not only a global crisis with regard to public health and economic stability, but also of family relationships and wellbeing (4). A detailed understanding of both physical and psychological longstanding consequences of school closures are of ongoing research interest [4,5,6,7]. Overall, school and kindergarten closures increase the need for family-provided childcare and represent a significant disruption to people’s daily lives and work–family arrangements, especially among working parents [8]. In particular, the disproportional burden on working mothers due to childcare as a consequence of the forced fusion of work and family and the demand to be available for both has garnered increasing public attention [9]. Described strategies to cope with the disruptive situation include rescheduling of daily activities and creative ways to care for others and themselves through social media connections [10]. However, besides intracouple division of childcare and gender disparities, little is known about other strategies to reconcile work and parenting as the pandemic progresses and fewer parents can rely on (un/paid) leave. Additionally, as the crisis lingers, so-called ‘pandemic fatigue’ associated with a gradual reduction from initial levels of adherence to protective behaviors and NPIs raises public health concerns [11]. This may be especially pronounced in vulnerable and burdened groups since parents experience higher levels of stress due to constant caregiving to children, economic and job-related worries, the elimination of the work–home boundary and lack of social support compared with nonparents [12].

In light of this, our exploratory study aimed to understand how families with two working parents or a single working parent (full- or part-time) organize childcare, homeschooling and work after a year in a pandemic when educational institutions are temporarily reclosed for the foreseeable future and close contact with others should be avoided and minimized. School closures are likely to be a widely imposed and recurrent NPI during the ongoing pandemic affecting a large proportion of society. It is thus essential to understand which strategies arise when childcare is repeatedly moved from institutions to the nuclear (working) family, as societal behavior has a central epidemiological impact. Our leading research questions were therefore:

(i) Who is taking care of the children during temporary lockdown situations within working families?
(ii) Does the shift of childcare into the private sphere without institutional support have any effects on the physical and mental wellbeing of the family members and might it have negative epidemiological consequences?

We collected data during one week of ‘hard lockdown’ in February–March 2021 (from 25 February 2021 to 1 March 2021) when schools, kindergartens and nurseries in Northern Italy for children of all ages were closed. During this week, so-called ‘emergency child care’ was only accessible for a small number of children with parents working in systemically important areas like the health sector. Additional NPIs for the relevant timeframe included a prohibitions on meeting people outside the nuclear family, receiving visits at home, and leaving the house except for work (systemically important sectors only), medical reasons, or situations of necessity or urgency [13]. In light of the fact that the pandemic due to SARS-CoV-2 remains an ongoing threat including genetic variants with potentially increased transmissibility and pathogenicity and since other threats will continue to be global challenges as both acute and chronic conditions are increasing due to environmental hazards, anthropogenic activities, and social disparities [14], the accurate understanding of how public health measures are implemented in an ongoing crisis by different groups is of high relevance for the development of resilient and realizable strategies of prevention.

### Aims

The present study aims to understand:

- Which work–family-arrangement strategies were adopted by working parents (two working parents or one single working parent) during a week of ‘hard lockdown’ in February 2021, when schools, kindergartens and nurseries were closed and individuals were not allowed to meet with people outside their nuclear family?
- How do parents describe their own and their children’s mental and physical health during the week of ‘hard lockdown’?

To the best of our knowledge, this is the first examination of working parents’ de facto applied coping strategies with regard to childcare within and outside the nuclear family in an advanced stage of the COVID-19 pandemic when childcare institutions were forced into temporary closure.

## 2. Materials and Methods

We performed a quantitative survey with a short online questionnaire for parents in the northernmost Italian region (Province of Bolzano) with a catchment area of 531,178 predominantly German-speaking inhabitants characterized by an autonomous political system. According to Italian law, approval by the Ethics Committee and written informed consent are not required in questionnaire-based and register-based population studies. The provision of information about the survey and its purpose, as well as voluntary participation provided implied consent. The study was performed in accordance with the Italian Personal Data Protection Law (Legislative Decree no. 196 of 30 June 2003) and was undertaken in accordance with the World Medical Association of Helsinki Declaration [15]. Consensus-based checklist recommendations for the reporting of survey studies (CROSS) were followed [16].

### 2.1. Questionnaire and Data Collection

Due to the novelty of the situation and the lack of standardized questionnaires adequately addressing our research questions, a short questionnaire was developed to create a small set of items collecting information about:

- coping strategies with regard to childcare and
- subjective evaluation of individuals‘ health status during the closure of childcare institutions.

The items were developed according to the literature on relevant prior research. The multiple choice questions focused on different coping behaviors (see supplementary file). The subjective evaluation of the participants’ own and their children’s health status constitutes the second thematic focus. Further data on demographics, family status, and living situation were collected. To limit the survey length in stressful times and since including a large number of different scales would have increased the length substantially, possibly resulting in a decreased response rate, we developed a 12-item scale including both closed-ended multiple choice items with nominal response options and 5-point Likert-type scales (see supplementary file). In order to check for adequate item variance, to ensure that respondents interpreted the items the manner that was intended, and to ensure that the language was unambiguous and easily understood, pilot testing with members of the target population was conducted. The survey was open for data collection only for a relatively short timeframe from 25 February 2021 to 1 March 2021, when schools, kindergarten and nurseries were de facto closed. Gathering data during an event minimizes recall errors and reduces known problems and pitfalls of retrospective surveys as respondents tend to give less accurate answers when asked about their past than when asked about their present [17]. However, the questionnaire included one retrospective item where participants were asked about their health and financial status before the outbreak of the pandemic. In order to minimize the cognitive effort associated with retrospective questions, the current scientific recommendations such as short and easy questions and using a specific anchor point were taken into account [18]. All questionnaires were digitally administered through Google Forms. The questionnaire was available in German and Italian.

### 2.2. Recruitment and Participants

In the study, only families with two working parents or a single working parent (full- or part-time) with children and youngsters up to age 18 living in one household were included. Snowball sampling, based on widespread posting on social media platforms, mostly within groups specific to parents, was used. Additionally, local newspapers raised awareness of the ongoing survey and childcare institutions shared the survey link with their clients. While non-probability sampling methods such as snowball sampling do not allow one to make representative inferences about a population, this method allowed us to conduct the survey within the relatively short timeframe of one week when schools and kindergartens were closed despite the lockdown with the reduced ability to reach out to participants via other forms of interaction.

### 2.3. Data Analysis

Data analysis was performed with IBM SPSS Statistics V26 (Property of IBM Corp., Torino, TO, Italy). Here, we present descriptive data of our primary survey, which led to the development of a comprehensive and representative study that we are currently conducting.

## 3. Results

### 3.1. Sample Description

The survey was open for data collection from 25 February 2021 to 1 March 2021—schools, kindergarten and nurseries were closed during this period—and within this time frame 3725 voluntary adult participants from different households responded (Table 1).

Overall, significantly more women (2449; 91%) filled in the questionnaire compared to men (350; 9%, chi square test, *p* < 0.01). Most households were composed of two adults (85%) with children. Single households with one adult only made up 10% of all cases and were composed mostly (395; 95.5%) of a female parent and child(ren). Slightly more than half of all participating households (55%) had two children under 18 years of age, 17% had three children, and 26% of all participating households had one child. Most children (65%) were between 6 and 11 years old (Table 2).

### 3.2. Who Is Watching the Children? Strategies during Lockdown

**Figure 1:**
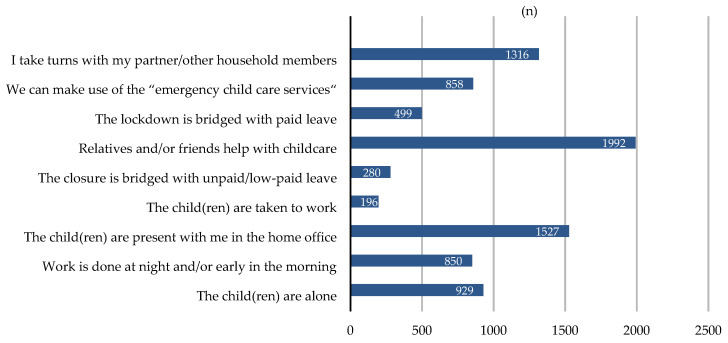
Who is watching the children? Working parents‘ most popular strategies for dealing with the closure of schools, kindergartens, and nurseries.

Within our cohort, slightly more than half of all participants (53%; n = 1992) sought help from people outside the nuclear family to bridge the gap and to care for their children. Single parents (24%; n = 60) more frequently sought help outside their kinship by asking friends and neighbors compared to families with two adults (15.5%; n = 324). In the vast majority of cases (79%; n = 1855), the grandparents took care of the children while schools, kindergartens, and nurseries were closed. In 16% (n = 387) of cases, non-relatives such as friends and neighbors helped the families with childcare. In 13% (n = 311) of cases, siblings took over the care for their younger brother and sisters. In families with three or more children in particular, siblings played an important role in compensating for missing childcare services (24.5%; n = 60). Within our cohort, 23% (n = 842) of all cases made use of so-called ‘emergency childcare’. However, while a large proportion activated contacts within or outside the family in order to guarantee childcare, many people found ways to provide childcare within the nuclear family; 35% (n = 1316) took alternating working–childcare-turns with their partners, 23% (n = 850) worked during nights and early in the morning (Figure 1). By the time of our survey (February/March 2021), eleven months after the first lockdown in Italy, only 13% (n = 499) were able to take paid leave or vacation to bridge the lockdown and 5% (n = 196) brought their children to their workplace. Additionally, 41% (n = 1527) had their children at their sides while working from home, and 25% (n = 292) left their children alone and unattended. Notably, compared to families with two adults (23.5%; n = 775), single parents in particular (37%; n = 141)—without the possibility to alternate with partners—left their children alone. It is worth mentioning that most families made use of multiple strategies, i.e., alternating shifts and involving other family members outside the core family.

With regard to the children’s age, parents are more likely to activate informal care support when the children are under 6 years; 70% of parents with children aged 0–6 years sought the help of relatives and friends to deal with the closure of childcare institutions, compared to 43% of families with children over 6 years. Likewise, the younger the children, the more work is done at night and early in the morning; 26.5% of parents work during these times within families with children <6 years compared to 18% within families with children > 6 years (Table 3). The older the children, the more often they are left alone (<6 years; 2%; >6 years; 41.1%).

### 3.3. Families under Pressure: Physical, Psychological and Financial Burden

Compared to the pre-pandemic situation, where only 5% (n = 179) found their families in a situation of psychological distress, during the time of our survey 63% (n = 2389) of all participating adults ranked their current psychological burden as ‘high’ or ‘very high’. Additionally, while only 4% (n = 143) of all participating parents reported physical distress before the pandemic, 27% (n = 1002) found themselves facing a high physical strain by February 2021. Regarding financial burdens, 21% (n = 780) found their families highly burdened, while only 2% claimed that this was the case prior to the pandemic (n = 58) (Table 4).

Regarding their children, the overall (physical and psychological) burden during lockdown followed a noticeable age-gradient; the older the children, the higher their burden and the lower their wellbeing as assessed by the parent—while at the same time parents have to fall back on less exhausting strategies (e.g., working during early/late hours; Table 5). On the other hand, the younger the children, the less stressed they are considered to be, but due to the higher intensity of care, parents more often have to rely on stressful coping strategies (e.g., working during early/late hours, taking turns, etc.).

## 4. Discussion

The COVID-19 pandemic has raised significant concerns regarding the effect of social disruptions on parental mental health, family well-being, and children’s adjustment. Due to the pace of the pandemic, measures of pandemic-related disruption and the behavioral response to NPIs over time have not been subject to rigorous empirical validation. We collected data during one week of ‘hard lockdown’ in February–March 2021 when schools, kindergartens and nurseries in Northern Italy for children of all ages were closed.

### 4.1. Adherence to NPIs and Pandemic Fatigue

Our results clearly illustrate that despite the government’s prohibition, a great proportion of working parents, seemingly left without other support systems, sought external help from third parties, initiating contact and thus being forced to shift childcare into the realm of ‘illegality’. This may be due to increasing ‘pandemic fatigue’, an inability to fall back on (paid) leave, and a decreased willingness to bear the burdens of an incompatible work–family situation at the expense of one’s own health and professional performance. However, conclusions about the course and decline of parental compliance with NPI measures are difficult to draw, since specific data for this target group within the first and second waves of COVID-19 are lacking. However, it is reasonable to assume that parents do not deliberately bypass legal frameworks due to low perceived personal risk for severe COVID-19, but rather because the individual cost–benefit analysis after over a year in a pandemic results in high costs and barriers and low personal benefits. Overall, social adherence to public health measures such as the closure of childcare institutions requires appropriate infrastructure and support systems for working parents.

### 4.2. Supported by the High Risk Group: Informal Childcare Arrangements during Lockdown

The fact that our cohort predominantly relied on help from the children’s grandparents deserves special attention, since by the end of February 2021 the elderly generation represented the unvaccinated high risk group; by the time of our survey only people within nursing homes and health personnel were vaccinated against SARS-CoV-2 in Northern Italy. Even though our results are not representative, the sole absolute number n = 1855 is an impressive epidemiological indication for various movements and social interactions during the week of ‘illegal’ contacts. Traditionally, grandparents play a fundamental role in providing informal childcare and thus increasing mothers’ labour market participation in Italy [19]. Against the background of socio-economic inequalities in the use of early infant care in Italy and the lack of private and public care of infants, family support systems substantially compensate for the patchy Italian coverage rate of formal childcare [20]. Informal childcare arrangements during the pandemic may, however, represent a major public health weakness, since the elderly are disproportionally affected by COVID-19 and informal contacts may be more difficult and time-consuming to trace in case of infections than, for example, contacts within the same kindergarten group with the same kindergarten teachers. Indeed, shifting childcare into the private sphere without support strategies, the use of coercive and punitive legal consequences, and judgmental rhetoric when working parents activate contacts outside the nuclear family may be counterproductive to public health goals since people may hesitate to report infections and contacts. Overall, childcare strategies during a crisis can hardly be understood without the cultural and formal framework conditions regarding childcare in “normal” times. Recently, a cross-country evaluation of seven European countries found that in all seven countries negative experiences of homeschooling prevailed; however, small differences between countries were found, with Italy being amongst the countries reporting the highest level of negative effect on parents [21]. Besides the different degrees of lockdown measures between countries, this may be due to the underlying cultural framework, role expectations, and lack of formal and informal care support systems. Overall, increasing childcare coverage during a pandemic clearly has a positive outcome on parental health and may also be a more effective prevention strategy with regard to the protection of the most vulnerable target group and fast and efficient contact tracing.

### 4.3. Informal vs. Formal Care

Informal family support systems are not available for all working parents. Our study indicates that other contacts, such as relatives, friends, and neighbors, were activated throughout the lockdown. Indeed, while the gaps in formal care in Italy gave rise to the development of a loosely regulated care market [22], our study indicates that only a few people (10%) within our cohort made use of private care providers, while most parents relied on informal care strategies. Besides financial constraints, role expectations, and lack of providers, additional explanations may be found in the unclear legal framework involving paid care providers during lockdown, since non-familial and external contacts were officially not allowed. The logistical stress of organizing informal care, along with the uncertainty and dependence on the arbitrariness and willingness of people within and outside the family to help, is likely to add to the burden of parents.

### 4.4. It’s All on ‘Mamma’: Gender Disparities and Intersectional Burden

Many studies have pointed out that the pandemic with its subsequent shutdown response has dramatically increased the care burden in women—who were already providing most of the world’s unpaid and mostly invisible care before the onset of the crisis [9]. To date, lockdown-associated gender disparities have been described by several studies and were not the central issue of our survey; however, it is worth noting that in response to our call it was mostly women who filled out the questionnaire. This resulted in a sample consisting of 90.5% female and only 9.2% male participants. This may be due to the fact that women in particular felt addressed by the topic. Overall, the gendered consequences of the pandemic intersect with other inequalities, leading to an increase in suboptimal strategies within the most vulnerable groups among parents such as single parents, migrants, low-income classes, or ethnic minorities. Working single parents—mostly women within our cohort—without the possibility to alternate with partners are, for instance, forced to leave their children unattended for longer periods compared to couples, as our study indicates. Thus far, few studies have indicated that single-adult households were impacted more negatively by the pandemic [23]. Overall, while gender disparities are well described, only limited data are currently available with regard to intersectional burden among parents (i.e., single parents, migrants, etc.) and further research is needed in order to understand and develop intervention strategies for the most vulnerable groups among parents.

### 4.5. A Lot of Trying and Combining: No Strategy Proves Optimal

Besides utilizing external help to care for them, children remain at their working parents’ side in home office situations, are unsupervised, or older siblings take care of the younger ones. None of these strategies appear optimal, since age dependent child-centered care cannot be assumed in any of these cases. In the case of two parents, models of alternating childcare turns, as well as nightshifts emerged as common coping strategies, but by February 2021 only a small proportion relied on strategies exclusively within their nuclear family. Our data points out that support strategies are necessary to guarantee both adequate childcare and protection from infection with SARS-CoV-2, since repeated lockdown situations cannot be handled by parents alone. Overall, the challenging organization of family life during lockdown did not pass without a trace, as our study and several others thus can attest to [24,25]. Youngsters and adolescents in particular, but also the parents themselves, appear psychologically and physically burdened. In accordance with previous studies where having younger children is associated with greater parenting-related exhaustion during lockdown [26], we found that parents are forced to rely on more strenuous strategies the younger their children are. With decreasing care intensity as they become older, parents more often leave them alone and use less external help. At the same time, older children and adolescents appear more burdened than younger children according to their parents.

Ultimately, the effective translation of public health measures into practicable strategies, the arising dynamics as a consequence of the implemented measures, as well as secondary and long-term damage due to repeated lockdown measures, are in need of further evaluation in order to develop resilient and tailored support strategies for those unable to fulfill public health measures.

### 4.6. Limitations of the Study

- Data were collected during the week of ‘hard lockdown’; however, parents and children were already aware that the lockdown would be eased and they would return to school and kindergarten the week after. This prospective knowledge might have influenced the perceived stress levels of our participants.
- Regarding the assessment of the family’s health status, we relied on parents’ retrospective judgement rather than collecting longitudinal data. Additionally, there is a lack of data regarding strategies for parenting and adherence to NPIs within the first lockdown for comparison.
- More female parents participated in our survey. Families with immigrant backgrounds, single parent households, and households with children with special needs, etc., are underrepresented.
- Within our cohort, we did not differentiate between the individual work situations of the parents, for instance smart working, full-time or part-time. Although it can be assumed that more working hours go hand in hand with higher pressure, solutions for children must also be found for those working few hours or doing part-time work or teleworking, since any working situation represents a largely untenable situation parallel to the closure of childcare institutions.
- Non-probability sampling methods such as snowball sampling do not allow us to make representative inferences about a population. Currently, we are conducting a representative follow-up study.

## 5. Conclusions

During the closure of schools and kindergartens, children of working parents are passed on to (changing) third parties for supervision. Ultimately, where the closure of institutions forcefully shifts and reduces childcare to the nuclear working family, new strategies including (i) health-damaging models of alternating work–childcare-shifts, (ii) ‘illegal’ involvement of, high-risks-groups and third parties outside the nuclear family, as well as (iii) neglect of age-related childcare arise as coping strategies. Our findings point out that working families need additional support strategies during repeated and even short lockdowns in order to be able to reduce contact, maintain their employment situation, and minimize secondary damage.

## Figures and Tables

**Table 1:** Sample description.

		n	%
**Language**	German	3305	86.5
	Italian	516	13.5
	Total	3821	100
**Gender**	Female	2449	90.5
	Male	350	9.2
	Divers	10	0.3
	Total	3809	100
**Household composition**	Single household (one adult with children)	386	10.1
	Two adults with children	3237	84.7
	More than two adults with children	146	3.8
	Other constellation	52	1.4
	Total	3821	100
**Single households**	Female parent	359	95.5
	Male parent	16	4.3
	Total	376	100
**Children under 18 years**	1	963	25.7
	2	2060	55.0
	3	627	16.7
	4	88	2.3
	5	7	0.2
	More than 5	3	0.1
	Total	3748	100
**Children** **’s age groups**	Families with children <6 years	797	21.3
	Families with children ≥6 years	1930	51.5
	Families with children <6 and >6 years	1020	27.2

**Table 2:** Children’s age in our cohort.

Children’s Age	n	%
>2 years	411	6.7
2 years	354	5.8
3–5 years	1452	23.6
6–11 years	2437	39.6
12–14 years	931	15.1
15–18 years	563	9.2

**Table 3:** Most popular coping strategies according to the children’s age.

	Families with Children <6 Years		Families with Children <6 and >6 Years		Families with Children ≥6 Years		Total	
	n	%	n	%	n	%	n	%
I take turns with my partner/other household members	333	42.4	363	36.5	596	31.8	1292	35.3
We can make use of the ‘emergency’ child care	220	28.0	273	27.4	349	18.6	842	23.0
The lockdown is bridged with paid leave	123	15.6	141	14.2	227	12.1	491	13.4
Relatives and/or friends help with childcare	549	69.8	606	60.9	801	42.7	1956	53.5
The closure is bridged with unpaid/low-paid leave	71	9.0	102	10.3	105	5.6	278	7.6
The child(ren) are taken to work	42	5.3	67	6.7	82	4.4	191	5.2
The child(ren) are present with me in the home office	284	36.1	448	45.0	765	40.8	1497	41
Work is done at night and/or early in the morning	208	26.5	283	28.4	342	18.2	833	22.8
The child(ren) are alone	14	1.8	126	12.7	771	41.1	911	24.9
Total	786	100	995	100	1874	100	3655	100

**Table 4:** Physical, psychological, and financial burden of working parents during the current lockdown and before the pandemic.

	None	Low	Medium	High	Very High
Current physical burden (during lockdown)	11% (n = 408)	26% (n = 967)	36.2% (n = 1349)	19.3% (n = 720)	7.6% (n = 282)
Estimated physical burden before the pandemic	22.6 (n = 847)	45% (n = 1688)	28.7% (n = 1076)	3.5% (n = 131)	0.3% (n = 12)
Current psychological burden (during lockdown)	2.3% (n = 86)	7.8% (n = 295)	26.5% (n = 1000)	35.4% (n = 1336)	27.9% (n = 1053)
Estimated psychological burden before the pandemic	15.7% (n = 594)	47.4% (n = 1790)	32.1% (n = 1212)	4.1% (n = 156)	0.6% (n = 23)
Current financial burden	25.9% (n = 962)	25.8 (n = 958)	27.4% (n = 1018)	12.8% (n = 477)	8.1% (n = 303)
Estimated financial burden before the pandemic	43.6% (n = 1620)	38.9% (n = 1444)	15.9% (n = 591)	1.4% (n = 52)	0.2% (n = 6)

**Table 5:** Children’s burden with regard to age group as assessed by their parents.

	None/Very Low		Medium		High/Very High		
	n	%	n	%	n	%	Total
0–2 years	468	61	197	26	105	14	770
3–5 years	595	41.2	570	39.5	279	19.3	1444
6–11 years	495	20.4	1049	43.3	878	36.6	2422
12–14 years	155	16.7	342	36.9	430	46.4	927
15–18 years	89	16.3	215	39.4	242	44.3	546
Total	1802	29.5	2373	38.8	1934	31.7	6109

## Data Availability

The data presented in this study are available on request from the corresponding author.

## Supplementary Materials

The questionnaire is available online at https://www.mdpi.com/article/10.3390/ijerph182111174/s1.
