# Supplementary material for: Who Is Watching the Children? A Quantitative Analysis of Strategies for Reconciling Work and Parenting during Lockdown in Northern Italy
Source: Int J Environ Res Public Health. 2021 Oct 24;18(21):11174. doi: 10.3390/ijerph182111174 (PMC8583403; doi:10.3390/ijerph182111174)
Supplement: Supplementary file 1 [file ijerph-18-11174-s001.zip › ijerph-1432206-supplementary.pdf]

**Survey on lockdown in South Tyrol (February 2021)**<br>**"Reconciliation of family and work" for families with children under 18 years of age**
This survey is addressed to families in which both parents are working or to working single parents in South Tyrol who have to take over the education and care of their children at home due to the closure of kindergartens, schools and nurseries.
What strategies have you found as a family to balance child care/education and work during lockdown? Please provide ALL the answers that apply to your situation:
A I take turns with my partner or other household members.
B My child/children can make use of the emergency care.
C The closure of the facilities is bridged with paid leave.
D Relatives and/or friends help to bridge the closures.
E Closure is bridged with unpaid/low-paid parental leave or another form of unpaid leave.
F The child/children are taken to work.
E The child/children are present in the home office.
H Work is done at night and/or early in the morning.
I The child/children are alone.
J No satisfactory solution has been found.
K Other (please specify in the comment field).
If you rely on the help of relatives and/or friends to bridge the situation: Who are they? More than one answer possible.
Grandparents and/or great-grandparents
Siblings
Other relatives
Friends/neighbours
Paid private childcare – paid babysitters

How would you rate the CURRENT burden on your CHILDREN (starting with the youngest)?
None
Low
Medium
High
Very High
How would you rate YOUR personal CURRENT burden (in lockdown)? Physical strain/ Psychological strain (e.g. stress)/Financial strain
None
Low
Medium
High
Very High
How would you rate YOUR personal burden BEFORE the Corona pandemic? Physical strain/ Psychological strain (e.g. stress)/Financial strain
None
Low
Medium
High
Very High

My household consists of ...
... two adults with child/children.
... more than two adults with child/children.
... one adult person with child/children.
... another situation (specify in the comment field if necessary).
Who do the current difficulties regarding the reconciliation of family and work in your family burden the most?
the woman
the man
both in equal shares
How many children under 18 do you have?
1
2
3
4
5
More than 5
How old are your children? More than one answer possible.
Under 2 years old
2 years old
3 - 5 years old

6 - 11 years old
12 - 14 years old
15 - 18 years old
You are...
a woman
a man
other response
Where do you live?
On a farm or in a home in the countryside
In a village in a rural region
In a suburb or village near the city
In a small town
In Bolzano (approx. 100.000 inhabitants)
In which part/valley of South Tyrol do you live?
Bozen/Bolzano
Burggrafenamt
Eisacktal
Pustertal
Salten-Schlern
Überetsch Unterland

Vinschgau
Wipptal
